# The emergence of new SARS-CoV-2 omicron subvariants introduces uncertainty about the end of the COVID-19 pandemic

**DOI:** 10.3389/fmed.2022.1010489

**Published:** 2022-10-10

**Authors:** Severino Jefferson Ribeiro da Silva

**Affiliations:** Department of Pharmaceutical Sciences, Leslie Dan Faculty of Pharmacy, University of Toronto, Toronto, ON, Canada

**Keywords:** SARS-CoV-2, pandemic, variants of concern, omicron, public health

## Background

In late December 2019, health authorities reported a cluster of patients with pneumonia of unknown cause that was epidemiologically linked to a seafood market in Wuhan, Hubei Province, China (1). The etiological agent was identified as a novel coronavirus, eventually named severe acute respiratory syndrome coronavirus 2 (SARS-CoV-2), and the respiratory illness was designated as coronavirus disease-2019 (COVID-19) by the World Health Organization (WHO) (2). Since its emergence, the rapid global spread of SARS-CoV-2 has provoked a catastrophic impact in our health and economic systems, causing a devastating pandemic and at the same time testing the resilience of the human population. As of September 5, 2022, more than 604.5 million cases of COVID-19 and 6.4 million deaths have been reported around the world. Most of the cases have been reported by the USA, followed by India, Brazil, and France (3).

The emergence of SARS-CoV-2 has been characterized by the identification of several variants of concern (VOCs) during the pandemic course: alpha (B.1.1.7), beta (B.1.351), gamma (P.1), and delta (B.1.617.2) (4, 5). Over the last 2 years these emerging variants have been associated with an abrupt increase in the number of COVID-19 cases, catalyzing several waves of the pandemic in many countries around the globe (6). More recently, the omicron (B.1.1.529) variant was first reported in Botswana and South Africa at the end of November 2021. After its emergence, this new variant initially named BA.1, spread rapidly across the world and was classified as a VOC by the WHO on 26 November 2021. The BA.1 subvariant rapidly spread around the world and outcompeted other SARS-CoV-2 variants, such as delta variant (7). In a rapidly moving field of study, a cumulative body of findings has demonstrated that the omicron variant is associated with high transmissibility and less severe illness in the human population, has resistance against most therapeutic antibodies, has robust binding to human ACE2 receptor, and may escape from neutralizing antibody responses in both convalescent and vaccinated individuals (8–11).

**FIGURE 1:**
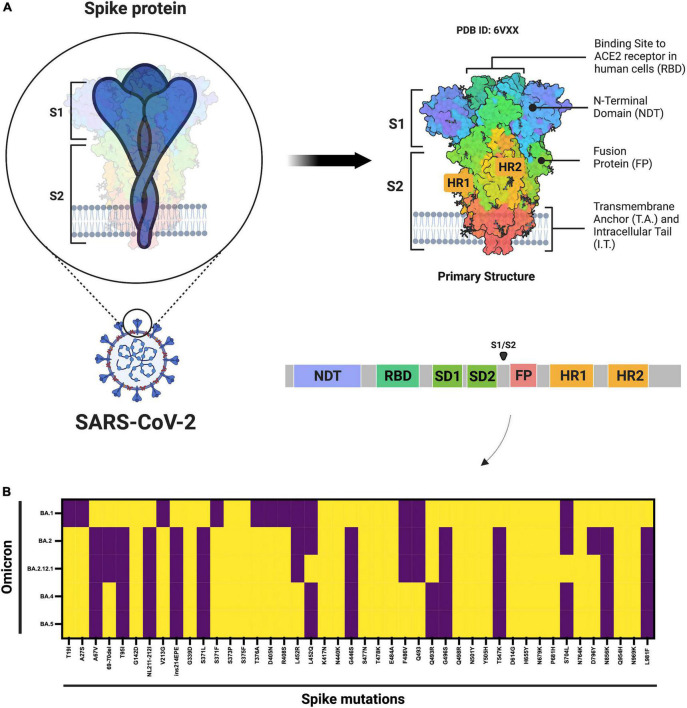
Schematic diagram of the SARS-CoV-2 spike protein and omicron subvariant mutations. **(A)** Shows the architecture of the SARS-CoV-2 spike protein. **(B)** Shows the mutations that have been identified in the omicron BA.1, BA.2, BA.2.12.1, and BA.4 or BA.5 subvariants. ACE2, angiotensin–converting enzyme 2; FP, fusion peptide; HR1. heptad repeat 1; HR2, heptad repeat 2; NTD, N-terminal domain; RBD, receptor-binding domain; SD1, subdomain 1; SD2, subdomain 2. Yellow denotes the presence, while purple denotes the absence of the specific mutation. The figure was created with Biorender.com.

However, in the past few months, multiple subvariants (BA.1.1, BA.2, BA.2.12.1, BA.3, BA. 4, and BA.5) of the omicron have emerged and raised great concerns to global health (12). These omicron subvariants carry a distinctive constellation of mutations, including several that have been previously determined to be of virological importance to other previous SARS-CoV-2 variants (Figure 1) (13). Among them, BA.2 has recently spread to many countries worldwide (14). Within a short time, research groups around the world have rapidly provided relevant insights about this novel omicron subvariant. Recent progress has shown that the effective reproduction number (R_0_) of BA.2 was 1.4-fold higher than that of BA.1 subvariant (7). Immunological studies demonstrated that the immunity induced by most COVID-19 vaccines administered to human populations is not effective against BA.2 subvariant (7). Collectively, *in vitro* and *in vivo* experiments showed that the BA.2 spike confers high capacity to replicate in human nasal epithelial cells and is more pathogenic than the BA.1, as demonstrated in hamsters (7).

As the COVID-19 pandemic progressed, other omicron subvariants are becoming protagonists (Table 1). BA.5 is currently the dominant subvariant in the USA, indicating that this subvariant may have selective advantages when compared to other SARS-CoV-2 omicron variants (15). Moreover, many countries around the world have documented cases associated with the circulation of these novel omicron subvariants.^1^ A recent report provided relevant immunological insights in terms of neutralizing antibody titers produced against the Wuhan virus along with omicron subvariants (BA.1, BA.2, BA.2.12.1, BA.4 or BA.5). In that study, Hachmann and colleagues conducted a study including 27 individuals who had been vaccinated and boosted with mRNA vaccine (BNT162b2) and 27 individuals who had been infected with the BA.1 or BA.2 subvariants (12). The results revealed that the omicron subvariants (BA.2.12.1, BA.4, and BA.5) substantially escape neutralizing antibodies induced by previous natural infection and vaccination. Interestingly, the immunological data demonstrated that the neutralizing antibody titers against the subvariants (BA.4, BA.5 or BA.2.12.1) was lower than antibody titers against the BA.1 and BA.2 omicron subvariants (12). Taken together, these findings indicate that the SARS-CoV-2 omicron variant has evolved multiple immune evasion strategies to escape from host the immune response for successful viral replication. This is exemplified due to fact that the omicron spike inefficiently utilizes the TMPRSS2 for cell entry via plasma membrane fusion. Instead, the omicron variant demonstrates a greater dependency on cell entry via the endocytic route (16).

The widespread dissemination of the SARS-CoV-2 omicron variant has been a devastating threat to pandemic control, indicating that we need to reconsider several features of the virus that had been previously thought to be established. After approximately 2 years and 5 months since the beginning of the pandemic, the emergence of new omicron subvariants introduces uncertainty about the end of the COVID-19 pandemic, at least for now. Although vaccine deployment has contributed to the reduction in the number of hospitalizations and deaths, many countries worldwide have experienced an abrupt increase in the number of COVID-19 cases in the past few months, catalyzing a new wave of the COVID-19 pandemic.

With this in mind, there are some other factors that the healthcare authorities can consider to declare the end of the COVID-19 pandemic: (i) disease severity and mortality due to new SARS-CoV-2 variants and they differ in vaccinated and non-vaccinated individuals. To address this question, recent findings suggested that alpha, beta, gamma, and delta SARS-CoV-2 variants are more serious than the Wuhan virus in terms of hospitalization, intensive care unit (ICU) admission, and mortality (17). In patients with the omicron VOC, the risk of hospitalization or death was considered lower when compared to infected patients with delta variant (18). However, it is important to consider that the high transmissibility and immune evasion properties of the omicron subvariants can lead to an increase in the rate of infection and mortality in older people with comorbidities, as we can see in the case of Japan and China after the emergence of the omicron subvariants (3, 19). While most SARS-CoV-2 variants are linked with breakthrough infections in fully vaccinated individuals, a cumulative body of data has shown that the vaccinated recipients showed a faster clearance time compared to non-vaccinated individuals (20) and reduced risk of death in patients infected with SARS-CoV-2 variants (21). Instead, unvaccinated individuals remained at the highest risk of infection, severe outcomes, and death (21). Therefore, continued efforts to increase vaccination and the establishment of booster campaigns in the human population are of paramount importance to provide protection and overcome the COVID-19 crisis. (ii) How about the authorized antiviral therapies against new omicron variants and comparative efficacy among vaccinated and non-vaccinated individuals. In this way, antiviral drugs and mass inoculations demonstrated a critical role in treating COVID-19 patients, especially to reduce the number of severe cases and deaths (22). According to the Food and Drug Administration (FDA), COVID-19-related therapies include antiviral drugs, immunomodulators, neutralizing antibody therapies, cell therapies, and gene therapies.^2^ Based on the ongoing and past pandemic control experiences, some antiviral drugs are questionable for the treatment of patients infected with the omicron VOC and for use in clinical practice (23). Recent studies have shown that the omicron VOC is resistant to most therapeutic monoclonal antibodies (mAbs) (13). In terms of antiviral drugs, recent reports have shown that nirmatrelvir, remdesivir, PF-0730481472, and molnupiravir are effective against infection with the omicron VOC, indicating that these options may be used for clinical practice for the treatment of patients (23, 24). More recently, multiple reports demonstrated that the PAXLOVID™ (oral tablets of nirmatrelvir and ritonavir, a SARS-CoV-2 protease inhibitor) was able to reduce the risk of hospitalization or death by 89% (within 3 days of symptom onset) and 88% (within 5 days of symptom onset) when compared to the placebo group (25). Despite efforts and recent advances, we need to revise and update frequently the therapeutic arsenal against the SARS-CoV-2 infection based on the past lessons, current experiences, and features of the omicron variant and other SARS-CoV-2 variants. As we have seen for other viruses (e.g., human immunodeficiency virus, HIV), if we create selective pressure on the virus, this can favor the emergence of mutations that help it to survive in the presence of the drug, especially in the case of use of protease inhibitor or viral life cycle inhibitor drugs.

**TABLE 1:** Characterization of SARS-CoV-2 omicron subvariants.

Omicron lineage	Emergence	Spike mutations	Transmissibility*^a^*	hACE2 binding	Disease severity	Resistance against therapeutic antibodies and neutralization by convalescent and vaccinated sera
BA.1	South Africa and Botswana, November 2021	A67V 69-70del T95I G142D NL211-212I ins214EPE G339D S371L S373P S375F K417N N440K G446S S477N T478K E484A Q493R G496S Q498R N501Y Y505H T547K D614G H655Y N679K P681H N764K D796Y N856K Q954H N969K L981F	Highly transmissible	Robust binding (13)	Less severe outcomes among infected individuals (11)	Resistance against most therapeutic monoclonal antibodies (mAbs) (26) Escape from neutralizing antibody responses in both convalescent and vaccinated individuals (13, 27)
BA.2	South Africa, December 2021	T19I A27S G142D V213G G339D S371F S373P S375F T376A D405N R408S K417N N440K S477N T478K E484A Q493R Q498R N501Y Y505H D614G H655Y N679K P681H N764K D796Y Q954H N969K	The effective reproduction number of BA.2 is 1.4-fold higher than that of BA.1 (7)	BA.2 spike is more fusogenic than BA.1 spike (7) Able to bind to mouse ACE2 with high potency (28)	BA.2 spike-bearing virus is more pathogenic than BA.1 (7)	BA.2 is resistant to BA.1-induced humoral immunity (7) Resistance against most therapeutic monoclonal antibodies (mAbs) (29)
BA.2.12.1	North-America, December 2021	T19I A27S G142D V213G G339D S371F S373P S375F T376A D405N R408S K417N N440K L452Q S477N T478K E484A Q493R Q498R N501Y Y505H D614G H655Y N679K P681H S704L N764K D796Y Q954H N969K	Highly transmissible (30)	BA.2.12.1 exhibits similarities in terms of ACE2-binding affinities to BA.2 (30)	No evidence yet of increased severity	BA.2.12.1 is more resistant (1.8-fold) to sera from vaccinated and boosted individuals than BA.2 (31) BA.2.12.1 and BA.4/BA.5 demonstrate stronger neutralization evasion than BA.2 against the plasma from boosted individuals (30)
BA.4	South Africa, January 2022	T19I A27S Del 69-70 G142D V213G G339D S371F S373P S375F T376A D405N R408S K417N N440K L452R S477N T478K E484A F486V Q498R N501Y Y505H D614G H655Y N679K P681H N764K D796Y Q954H N969K	Highly transmissible (30)	BA.4 exhibits similarities in terms of ACE2-binding affinities to BA.2 (30)	No evidence yet of increased severity	Reduced neutralization by serum from triple AstraZeneca or Pfizer vaccinated individual (32) Reduced activity of SARS-CoV-2 therapeutic antibodies against BA.4 (32) More likely to lead to vaccine breakthrough infections among individuals (31) BA.4 demonstrate stronger neutralization evasion than BA.2 against the plasma from boosted individuals (30)
BA.5	South Africa, January 2022	T19I A27S Del 69-70 G142D V213G G339D S371F S373P S375F T376A D405N R408S K417N N440K L452R S477N T478K E484A F486V Q498R N501Y Y505H D614G H655Y N679K P681H N764K D796Y Q954H N969K	Highly transmissible (30)	BA.5 exhibits similarities in terms of ACE2-binding affinities to BA.2 (30)	No evidence yet of increased severity	Reduced neutralization by serum from triple AstraZeneca or Pfizer vaccinated individual (32) Reduced activity of SARS-CoV-2 therapeutic antibodies against BA.5 (32) More likely to lead to vaccine breakthrough infections among individuals (31) BA.5 demonstrate stronger neutralization evasion than BA.2 against the plasma from boosted individuals (30)

^*a*^Viral zone (https://viralzone.expasy.org/9556).

## Final considerations and public health perspectives

**FIGURE 2:**
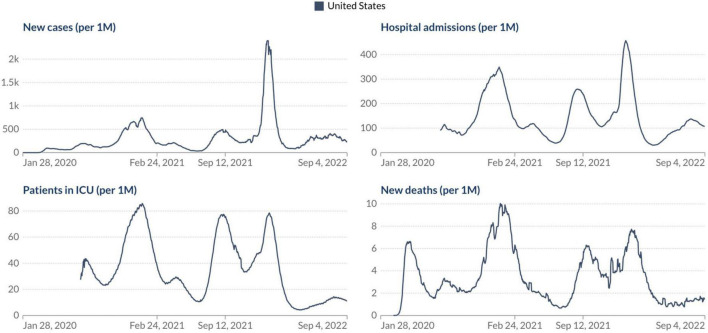
Epidemiological situation of the COVID-19 pandemic in the USA. Johns Hopkins University CSSE COVID-19 Data (https://ourworldindata.org/).

Based on our current scenario, what should we expect from our future with SARS-CoV-2? As WHO Director General Dr. Tedros Adhanom Ghebreyesus said, “the pandemic is far from over – and it will not be over anywhere until it’s over everywhere.” Considering all these factors discussed above appears we must take a step back regarding the relaxation of COVID-19 control measures and indicate that it is not yet time to let our guard down in the face of this devastating virus, especially after the emergence of the omicron subvariants. But the good news is if we look at the current epidemiological scenario in the USA, the most affected country during the COVID-19 pandemic. Analyzing the number of confirmed COVID-19 cases, deaths, hospital admissions, and patients in ICU per million people it is evident that we can see a light at the end of the tunnel, but we still have a considerable way to go to see the end of the pandemic phase (Figure 2). What should we expect in the coming months? Different countries around the world will experience the coming phase differently based on some critical factors: vaccine coverage, availability and application of boosters in the human population, dynamics of seasonality, demographics, government policies, and implementation of strategies to reduce the transmission of the virus.

Notably, the SARS-CoV-2 omicron variant will probably not be the last VOC, which suggests that we should prepare for the emergence of new variants that can lead to further immune evasion and render current vaccine ineffective over time. The administration of booster shots using mRNA vaccines as an extra layer of protection will be of paramount importance to overcome the COVID-19 pandemic and combat the impact of further emerging SARS-CoV-2 variants. As with flu, it appears that we should monitor and update the composition of COVID-19 vaccines frequently, as COVID-19 may continue to be an endemic disease in the world. At the same time, we need to learn to live the new “normal” lifestyle with the hope that SARS-CoV-2 does not bring serious concerns to global health in the near future.

## Author contributions

SS conceived the work, wrote the original draft, and reviewed the final manuscript.

## Footnotes

1
https://www.gisaid.org/hcov19-variants/

2
https://www.fda.gov/drugs/coronavirus-covid-19-drugs/coronavirus-treatment-acceleration-program-ctap

## References

[1] Zhu N Zhang D Wang W Li X Yang B Song J A novel coronavirus from patients with pneumonia in China, 2019. *N Engl J Med.* (2020) 382:727–33. 10.1056/NEJMoa2001017 31978945 PMC7092803

[2] Rambaut A Holmes EC O’Toole Á Hill V McCrone JT Ruis C A dynamic nomenclature proposal for SARS-CoV-2 lineages to assist genomic epidemiology. *Nat Microbiol.* (2020) 5:1403–7. 10.1038/s41564-020-0770-5 32669681 PMC7610519

[3] Dong E Du H Gardner L. An interactive web-based dashboard to track COVID-19 in real time. *Lancet Infect Dis.* (2020) 20:533–4. 10.1016/S1473-3099(20)30120-1 32087114 PMC7159018

[4] da Silva SJR de Lima SC da Silva RC Kohl A Pena L. Viral load in COVID-19 patients: implications for prognosis and vaccine efficacy in the context of emerging SARS-CoV-2 variants. *Front Med (Lausanne).* (2021) 8:836826. 10.3389/fmed.2021.836826 35174189 PMC8841511

[5] Silva SJRD Pena L. Collapse of the public health system and the emergence of new variants during the second wave of the COVID-19 pandemic in Brazil. *One Health.* (2021) 13:100287. 10.1016/j.onehlt.2021.100287 34222607 PMC8240439

[6] da Silva SJR do Nascimento JCF Germano Mendes RP Guarines KM Targino Alves da Silva C da Silva PG Two years into the COVID-19 pandemic: lessons learned. *ACS Infect Dis.* (2022) 8:1758–814. 10.1021/acsinfecdis.2c00204 35940589 PMC9380879

[7] Yamasoba D Kimura I Nasser H Morioka Y Nao N Ito J Virological characteristics of the SARS-CoV-2 omicron BA.2 spike. *Cell.* (2022) 185:2103–15.e19. 10.1016/j.cell.2022.04.035 35568035 PMC9057982

[8] Silva SJRD Kohl A Pena L Pardee K. Recent insights into SARS-CoV-2 omicron variant. *Rev Med Virol.* (2022) e2373. 10.1002/rmv.2373 35662313 PMC9347414

[9] Garcia-Beltran WF Lam EC St Denis K Nitido AD Garcia ZH Hauser BM Multiple SARS-CoV-2 variants escape neutralization by vaccine-induced humoral immunity. *Cell.* (2021) 184:2372–83.e9. 10.1016/j.cell.2021.03.013 33743213 PMC7953441

[10] Planas D Saunders N Maes P Guivel-Benhassine F Planchais C Buchrieser J Considerable escape of SARS-CoV-2 omicron to antibody neutralization. *Nature.* (2021). 602:671–75. 10.1038/s41586-021-04389-z 35016199

[11] Nyberg T Ferguson NM Nash SG Webster HH Flaxman S Andrews N Comparative analysis of the risks of hospitalisation and death associated with SARS-CoV-2 omicron (B.1.1.529) and delta (B.1.617.2) variants in England: a cohort study. *Lancet.* (2022) 399:1303–12. 10.1016/S0140-6736(22)00462-7 35305296 PMC8926413

[12] Hachmann NP Miller J Collier AY Ventura JD Yu J Rowe M Neutralization escape by SARS-CoV-2 omicron subvariants BA.2.12.1, BA.4, and BA.5. *N Engl J Med.* (2022) 387:86–8. 10.1056/NEJMc2206576 35731894 PMC9258748

[13] Hoffmann M Krüger N Schulz S Cossmann A Rocha C Kempf A The Omicron variant is highly resistant against antibody-mediated neutralization: implications for control of the COVID-19 pandemic. *Cell.* (2021). 185:447–56. 10.1016/j.cell.2021.12.032 35026151 PMC8702401

[14] Yu J Collier AY Rowe M Mardas F Ventura JD Wan H Neutralization of the SARS-CoV-2 omicron BA.1 and BA.2 variants. *N Engl J Med.* (2022) 386:1579–80. 10.1056/NEJMc2201849 35294809 PMC9006770

[15] CDC. *Monitoring Variant Proportions.* Atlanta, GA: CDC (2022).

[16] Meng B Abdullahi A Ferreira IATM Goonawardane N Saito A Kimura I Altered TMPRSS2 usage by SARS-CoV-2 omicron impacts tropism and fusogenicity. *Nature.* (2022) 603:706–14. 10.1038/s41586-022-04474-x 35104837 PMC8942856

[17] Ong SWX Chiew CJ Ang LW Mak TM Cui L Toh MPHS Clinical and virological features of SARS-CoV-2 variants of concern: a retrospective cohort study comparing B.1.1.7 (Alpha), B.1.315 (Beta), and B.1.617.2 (Delta). *Clin Infect Dis.* (2021). 75:e1128–36. 10.1093/cid/ciab721 34423834 PMC8522361

[18] Ulloa AC Buchan SA Daneman N Brown KA. Estimates of SARS-CoV-2 omicron variant severity in Ontario, Canada. *JAMA.* (2022) 327:1286–8. 10.1001/jama.2022.2274 35175280 PMC8855311

[19] Zhang X Zhang W Chen S. Shanghai’s life-saving efforts against the current omicron wave of the COVID-19 pandemic. *Lancet.* (2022) 399:2011–2. 10.1016/S0140-6736(22)00838-8 35533708 PMC9075855

[20] Kissler SM Fauver JR Mack C Tai CG Breban MI Watkins AE Viral dynamics of SARS-CoV-2 variants in vaccinated and unvaccinated persons. *N Engl J Med.* (2021) 385:2489–91. 10.1056/NEJMc2102507 34941024 PMC8693673

[21] Cohn BA Cirillo PM Murphy CC Krigbaum NY Wallace AW. SARS-CoV-2 vaccine protection and deaths among US veterans during 2021. *Science.* (2022) 375:331–6. 10.1126/science.abm0620 34735261 PMC9836205

[22] Barnette K Gordon MS Rodriguez D Gary Bird T Skolnick A Schnaus M Oral sabizabulin for high-risk, hospitalized adults with Covid-19: interim analysis. *NEJM Evid.* (2022). 1:1–11. 10.1056/EVIDoa2200145 38319812

[23] Li P Wang Y Lavrijsen M Lamers MM de Vries AC Rottier RJ SARS-CoV-2 omicron variant is highly sensitive to molnupiravir, nirmatrelvir, and the combination. *Cell Res.* (2022) 32:322–4. 10.1038/s41422-022-00618-w 35058606 PMC8771185

[24] Takashita E Kinoshita N Yamayoshi S Sakai-Tagawa Y Fujisaki S Ito M Efficacy of antibodies and antiviral drugs against Covid-19 omicron variant. *N Engl J Med.* (2022) 386:995–8. 10.1056/NEJMc2119407 35081300 PMC8809508

[25] Hammond J Leister-Tebbe H Gardner A Abreu P Bao W Wisemandle W Oral nirmatrelvir for high-risk, nonhospitalized adults with Covid-19. *N Engl J Med.* (2022) 386:1397–408. 10.1056/NEJMoa2118542 35172054 PMC8908851

[26] Cameroni E Bowen JE Rosen LE Saliba C Zepeda SK Culap K Broadly neutralizing antibodies overcome SARS-CoV-2 Omicron antigenic shift. *Nature.* (2021). 602:664–70. 10.1038/s41586-021-04386-2 35016195 PMC9531318

[27] Garcia-Beltran WF St Denis KJ Hoelzemer A Lam EC Nitido AD Sheehan ML mRNA-based COVID-19 vaccine boosters induce neutralizing immunity against SARS-CoV-2 Omicron variant. *Cell.* (2022) 185:457–66.e4. 10.1016/j.cell.2021.12.033 34995482 PMC8733787

[28] Xu Y Wu C Cao X Gu C Liu H Jiang M Structural and biochemical mechanism for increased infectivity and immune evasion of omicron BA.2 variant compared to BA.1 and their possible mouse origins. *Cell Res.* (2022) 32:609–20. 10.1038/s41422-022-00672-4 35641567 PMC9152305

[29] Andreano E Paciello I Marchese S Donnici L Pierleoni G Piccini G Anatomy of omicron BA.1 and BA.2 neutralizing antibodies in COVID-19 mRNA vaccinees. *Nat Commun.* (2022) 13:3375. 10.1038/s41467-022-31115-8 35697673 PMC9189263

[30] Cao Y Yisimayi A Jian F Song W Xiao T Wang L BA.2.12.1, BA.4 and BA.5 escape antibodies elicited by omicron infection. *Nature.* (2022) 608:593–602. 10.1038/s41586-022-04980-y 35714668 PMC9385493

[31] Wang Q Guo Y Iketani S Li Z Mohri H Wang M SARS-CoV-2 omicron BA.2.12.1, BA.4, and BA.5 subvariants evolved to extend antibody evasion. *bioRxiv* [Preprint]. (2022). 10.1038/s41586-022-05053-w 35790190 PMC9385487

[32] Tuekprakhon A Nutalai R Dijokaite-Guraliuc A Zhou D Ginn HM Selvaraj M Antibody escape of SARS-CoV-2 omicron BA.4 and BA.5 from vaccine and BA.1 serum. *Cell.* (2022) 185:2422–33.e13. 35772405 10.1016/j.cell.2022.06.005 PMC9181312

